# 1463. Risk Factors for Primary Invasive Surgical Site Infections among Single Adult Liver Transplants at Duke University Hospital in the Period 2015-2019.

**DOI:** 10.1093/ofid/ofad500.1300

**Published:** 2023-11-27

**Authors:** Manuela Carugati, Sana Arif, Lindsay Y King, Matt Harris, Kayla B Evans, Andrew Barbas, Debra Sudan, Rachel Miller, Barbara D Alexander

**Affiliations:** Duke University, Durham, North Carolina; Duke University, Durham, North Carolina; Duke University, Durham, North Carolina; Duke, Durham, North Carolina; UF Health Shands, Gainesville, Florida; Duke University, Durham, North Carolina; Duke University, Durham, North Carolina; Duke University, Durham, North Carolina; Duke University School of Medicine, Durham, North Carolina

## Abstract

**Background:**

Invasive primary surgical site infections (IP-SSI) are a severe complication of liver transplant. Identification of risks for IP-SSI is critical to IP-SSI prevention.

**Figure:**
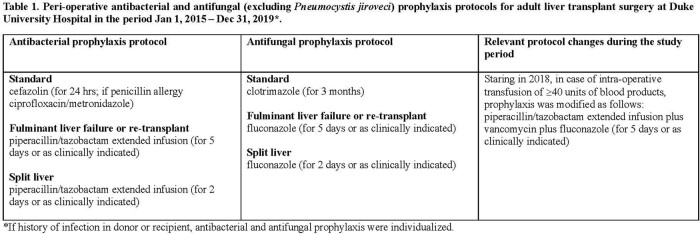

**Methods:**

All adult single liver transplants performed at Duke University Hospital in 2015-2019 were reviewed for IP-SSI occurring within 3 months of transplant. Antimicrobial prophylaxis regimens are noted in Table 1. Risks for IP-SSI were identified via backward stepwise multiple logistic regression using an entry cut-off of 0.01. Repeat transplantation, split liver, transplant surgery >8 hrs, Roux-en-Y biliary anastomosis, anastomotic leak, and repeat abdominal surgery within 3 months were entered into the initial model. Prior hepato-biliary surgery, pre-transplant immunosuppressive therapy, and living donor status were excluded due to potential collinearity with “repeat transplantation” and “split liver”. A two-sided p-value of < 0.05 was considered statistically significant.

**Figure:**
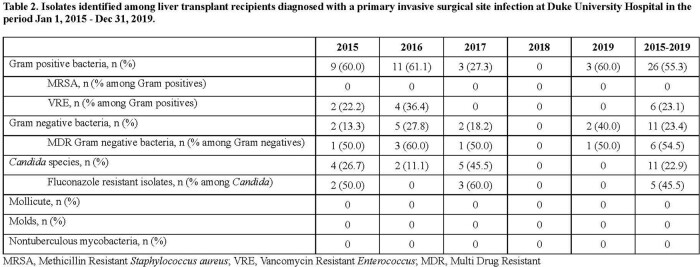

**Results:**

Among 380 single adult liver transplants performed, 25 (6.6%) IP-SSI were identified. IP-SSI were associated with longer hospital stay (25.0 vs. 9.0 days, p < 0.01). Among IP-SSI, 13 (52.0%) were polymicrobial and 12 (48.0%) monomicrobial. IP-SSI microbiology is detailed in Table 2. Fluconazole resistance occurred in 5 (45.5%) *Candida* isolates among which 3 (60.0%) broke through individualized echinocandin prophylaxis. Baseline recipient characteristics are shown in Table 3. Per Table 4, repeat transplantation, Roux-en-Y biliary anastomosis, anastomotic leak, and repeat abdominal surgery were significantly associated with IP-SSI risk.

**Table 3.**
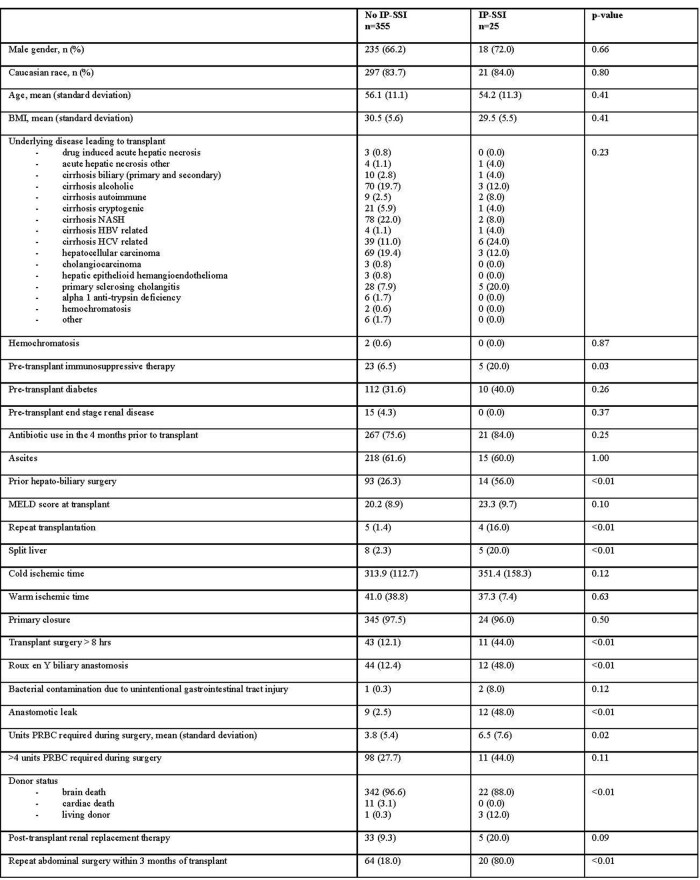
Baseline characteristics of adult patients who underwent a single liver transplant at Duke University Hospital in the period 1 Jan 2015 - 31 Dec 2019 stratified by the diagnosis of invasive primary surgical site infection (IP-SSI) within 90 days of transplant surgery. BMI, body mass index; HBV, hepatitis b virus; HCV, hepatitis c virus; MELD, model for end-stage liver disease; NASH, non-alcoholic steatohepatitis; PRBC, packed red blood cells

**Figure:**
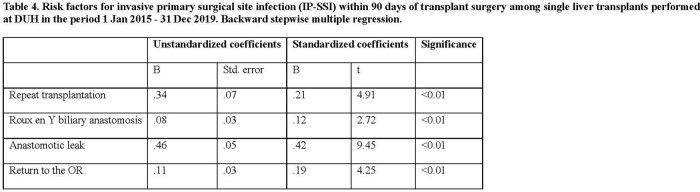

**Conclusion:**

The overall IP-SSI rate in our cohort is low compared to published literature supporting effectiveness of current antimicrobial prophylaxis. In this setting, factors that portend risk for IP-SSI are related to the surgical procedures, thus are not easily modifiable. Patients with repeat transplantation, Roux-en-Y biliary anastomosis, anastomotic leak, and repeat abdominal surgery within 3 months of transplantation must be monitored closely for development of IP-SSI. However, the appropriateness of current prophylaxis regimens should be further evaluated given the high prevalence of drug-resistant pathogens causing breakthrough IP-SSI.

**Disclosures:**

**Matt Harris, PharmD, MHS**, wolters kluer: Advisor/Consultant **Barbara D. Alexander, MD**, F2G Pharmaceuticals: Advisor/Consultant|HealthTrackRx: Advisor/Consultant|HealthTrackRx: Board Member|Leadiaint: Grant/Research Support|Merck: Advisor/Consultant|Scynexis: Grant/Research Support|Thermofisher: Advisor/Consultant

